# ASEPTIC: primary antibiotic prophylaxis using co-trimoxazole to prevent SpontanEous bacterial PeritoniTIs in Cirrhosis—study protocol for an interventional randomised controlled trial

**DOI:** 10.1186/s13063-022-06727-6

**Published:** 2022-09-27

**Authors:** Dominic Crocombe, Norin Ahmed, Indran Balakrishnan, Ekaterina Bordea, Marisa Chau, Louise China, Lynsey Corless, Victoria Danquah, Hakim-Moulay Dehbi, John F. Dillon, Ewan H. Forrest, Nick Freemantle, David Peter Gear, Coral Hollywood, Rachael Hunter, Tasheeka Jeyapalan, Yiannis Kallis, Stuart McPherson, Iulia Munteanu, Jim Portal, Paul Richardson, Stephen D. Ryder, Amandeep Virk, Gavin Wright, Alastair O’Brien

**Affiliations:** 1 UCL Institute of Liver and Digestive Health, Sheila Sherlock Liver Centre, Royal Free London NHS Foundation Trust, London, UK; 2 University College London Comprehensive Clinical Trials Unit, London, UK; 3 Royal Free London NHS Foundation Trust, University College London, London, UK; 4 Hull University Teaching Hospitals NHS Trust, Hull, UK; 5 Division of Molecular and Clinical Medicine, School of Medicine, University of Dundee, Dundee, UK; 6 Gastroenterology Unit, Glasgow Royal Infirmary, University of Glasgow, Glasgow, UK; 7 Cancer Research UK and UCL Cancer Trials Centre, London, UK; 8 Gloucestershire Hospitals NHS Foundation Trust, Gloucester, UK; 9 The Blizard Institute, Queen Mary University of London, London, UK; 10 Liver Unit, The Newcastle Upon Tyne Hospitals NHS Foundation Trust, The Translational and Clinical Research Institute, Newcastle University, Newcastle upon Tyne, UK; 11 University Hospitals Bristol NHS Foundation Trust, Bristol, UK; 12 Liverpool University Hospitals NHS Foundation Trust, Liverpool, UK; 13 NIHR Nottingham Biomedical Research Centre at Nottingham University Hospitals NHS Trust, University of Nottingham, Nottingham, UK; 14 Mid & South Essex NHS Foundation Trust, Basildon, UK; 15 University College London Hospitals NHS Foundation Trust, London, UK

**Keywords:** Liver cirrhosis, Ascites, Co-trimoxazole, Infection, Spontaneous bacterial peritonitis, Antimicrobial resistance, Primary prophylaxis

## Abstract

**Background:**

Bacterial infection is a major cause of mortality in patients with cirrhosis. Spontaneous bacterial peritonitis (SBP) is a serious and common infection in patients with cirrhosis and ascites. Secondary prophylactic antibiotic therapy has been shown to improve outcomes after an episode of SBP but primary prophylaxis to prevent the first episode of SBP remains contentious. The aim of this trial is to assess whether primary antibiotic prophylaxis with co-trimoxazole improves overall survival compared to placebo in adults with cirrhosis and ascites.

**Methods:**

The ASEPTIC trial is a multicentre, placebo-controlled, double-blinded, randomised controlled trial (RCT) in England, Scotland, and Wales. Patients aged 18 years and older with cirrhosis and ascites requiring diuretic treatment or paracentesis, and no current or previous episodes of SBP, are eligible, subject to exclusion criteria. The trial aims to recruit 432 patients from at least 30 sites. Patients will be randomised in a 1:1 ratio to receive either oral co-trimoxazole 960 mg or an identical placebo once daily for 18 months, with 6 monthly follow-up visits thereafter (with a maximum possible follow-up period of 48 months, and a minimum of 18 months). The primary outcome is overall survival. Secondary outcomes include the time to the first incidence of SBP, hospital admission rates, incidence of other infections (including *Clostridium difficile*) and antimicrobial resistance, patients’ health-related quality of life, health and social care resource use, incidence of cirrhosis-related decompensation events, liver transplantation, and treatment-related serious adverse events.

**Discussion:**

This trial will investigate the efficacy, safety, and cost-effectiveness of co-trimoxazole for patients with liver cirrhosis and ascites to determine whether this strategy improves clinical outcomes. Given there are no treatments that improve survival in decompensated cirrhosis outside of liver transplant, if the trial has a positive outcome, we anticipate widespread adoption of primary antibiotic prophylaxis.

**Trial registration:**

ClinicalTrials.gov NCT043955365. Registered on 18 April 2020. Research ethical approval was granted by the Research Ethics Committee (South Central – Oxford B; REC 19/SC/0311) and the Medicines and Healthcare products Regulatory Agency (MHRA).

## Administrative information

Note: The numbers in curly brackets in this protocol refer to the SPIRIT checklist item numbers [1]. The order of the items has been modified to group similar items (see http://www.equator-network.org/reporting-guidelines/spirit-2013-statement-defining-standard-protocol-items-for-clinical-trials/).

**Table:** 

Title {1}	ASEPTIC: Primary Antibiotic prophylaxis using co-trimoxazole to prevent SpontanEous bacterial PeritoniTIs in Cirrhosis—study protocol for an interventional randomised controlled trial
Trial registration {2a and 2b}	EudraCT # 2019–000,581-38 IRAS: 262,176 REC # 19/SC/0311
Protocol version {3}	Version 5.0, 20^th^ August 2021
Funding {4}	National Institute of Health and Care Research (NIHR) HTA grant number 17/67/01. No other external funding.
Author details {5a}	Dominic Crocombe (UCL Institute of Liver and Digestive Health; Sheila Sherlock Liver Centre, Royal Free London NHS Foundation Trust), Norin Ahmed (University College London Comprehensive Clinical Trials Unit), Indran Balakrishnan (Royal Free London NHS Foundation Trust; University College London), Ekaterina Bordea (University College London Comprehensive Clinical Trials Unit), Marisa Chau (University College London Comprehensive Clinical Trials Unit), Louise China (UCL Institute of Liver and Digestive Health; Sheila Sherlock Liver Centre, Royal Free London NHS Foundation Trust), Lynsey Corless (Hull University Teaching Hospitals NHS Trust), Victoria Danquah (University College London Comprehensive Clinical Trials Unit), Hakim-Moulay Dehbi (University College London Comprehensive Clinical Trials Unit), John F Dillon (Division of Molecular and Clinical Medicine, School of Medicine, University of Dundee), Ewan H Forrest (Gastroenterology Unit, Glasgow Royal Infirmary; University of Glasgow), Nick Freemantle (University College London Comprehensive Clinical Trials Unit), David Peter Gear (Cancer Research UK and UCL Cancer Trials Centre), Coral Hollywood (Gloucestershire Hospitals NHS Foundation Trust), Rachael Hunter (University College London Comprehensive Clinical Trials Unit), Tasheeka Jeyapalan (University College London Comprehensive Clinical Trials Unit), Yiannis Kallis (The Blizard Institute, Queen Mary University of London), Stuart McPherson (Liver Unit, The Newcastle upon Tyne Hospitals NHS Foundation Trust; The Translational and Clinical Research Institute, Newcastle University, Newcastle upon Tyne, UK), Iulia Munteanu (University College London Comprehensive Clinical Trials Unit), Jim Portal (University Hospitals Bristol NHS Foundation Trust), Paul Richardson (Liverpool University Hospitals NHS Foundation Trust), Stephen D Ryder (NIHR Nottingham Biomedical Research Centre at Nottingham University Hospitals NHS Trust; University of Nottingham), Amandeep Virk (University College London Comprehensive Clinical Trials Unit), Gavin Wright (Mid & South Essex NHS Foundation Trust), Alastair O’Brien (University College London Comprehensive Clinical Trials Unit; UCL Institute of Liver and Digestive Health; Sheila Sherlock Liver Centre, Royal Free London NHS Foundation Trust; University College London Hospitals NHS Foundation Trust)
Name and contact information for the trial sponsor {5b}	University College London (UCL) ctu.aseptic@ucl.ac.uk
Role of sponsor {5c}	University College London (UCL) is the trial sponsor and has delegated the sponsor responsible for the overall management of the ASEPTIC trial to the UCL Comprehensive Clinical Trials Unit (CCTU). Specific functions delegated to the UCL CCTU include a clinical project manager at UCL CCTU overseeing the trial manager, who will be responsible for the day-to-day management of the trial and providing support to the site staff. The CCTU will be involved in approaching sites, initiation visits, case report form development, database construction, and protocol and patient information development in collaboration with the Trial Management Group (TMG), Independent Data Monitoring Committee (IDMC), and Trial Steering Committee (TSC).

## Introduction

### Background and rationale {6a}

For patients with advanced liver disease, bacterial infection is a major cause of morbidity and mortality. In patients with cirrhosis and ascites, spontaneous bacterial peritonitis (SBP) is the most common serious infection [2, 3], and secondary prophylaxis with antibiotic therapy is routinely prescribed to prevent further infection in patients who recover from SBP [4–6]. Yet, despite 90% of SBP cases presenting in patients with no previous episode [7], there is considerable uncertainty regarding the use of antibiotic therapy to prevent SBP in patients with ascites and no history of SBP, primary prophylaxis. An episode of SBP triggers a long-term reduction in survival and quality of life in cirrhosis, and therefore, primary prophylaxis to prevent this in the first place may actually offer greater gain than secondary.

Studies have shown that patients with ascites who have a low protein content (< 1.5 g/dL) were associated with a higher risk of developing infection [8–10], possibly due to reduced host opsonisation activity [8, 11, 12]. However, more recently, 2 large-scale, post hoc analyses showed no association between low protein count and increased SBP risk [13, 14]. Both the National Institute of Health and Care Excellence (NICE) and the European Association for the Study of the Liver (EASL) recommend antibiotic prophylaxis with quinolones for patients with ascitic fluid concentration < 1.5 g/dL, but the evidence to support this is limited and dated [6, 15, 16]. The American Association for the Study of Liver Disease (AASLD) recommends primary prophylaxis (with norfloxacin or co-trimoxazole) only if the ascitic protein is < 1.5 g/dL in the presence of impaired renal function and liver failure (Child–Pugh score > 9 and bilirubin > 3 mg/dL), which accounts for very few patients [17]. Conversely, the British Society of Gastroenterology (BSG) limits its guidance on primary prophylaxis due to a lack of consensus [18].

With support from the BSG trial development group, we conducted a national survey on the use of primary prophylaxis for SBP, and responses were received from 23 NHS Trusts (unpublished data). Nine centres reported routine use of antibiotics for primary prophylaxis against SBP; for 7 other centres, this was not routine practice, and the remaining 7 reported variable practice differing between clinicians. Evidently, there is considerable uncertainty and therefore great need for robust evidence to guide primary prophylaxis, especially for patients with ascites and ascitic protein > 1.5 g/dL and less advanced liver disease, and regarding the optimal duration of therapy. This is important as no therapies exist that prolong life in these patients outside of liver transplantation, which only occurs in a minority [19, 20].

### Objectives {7}

The primary objective of this trial is to assess the effect of primary antibiotic prophylaxis with co-trimoxazole on overall survival compared to placebo in adults with cirrhosis and ascites, utilising a treatment policy estimand. Key secondary objectives include assessing the incidence of SBP, hospital admissions, *Clostridium difficile* (*C. difficile*)-associated diarrhoea and antimicrobial resistance, cost-effectiveness, and incidence of cirrhosis-related events, liver transplantation and treatment-related serious adverse events.

### Trial design {8}

**Fig. 1:**
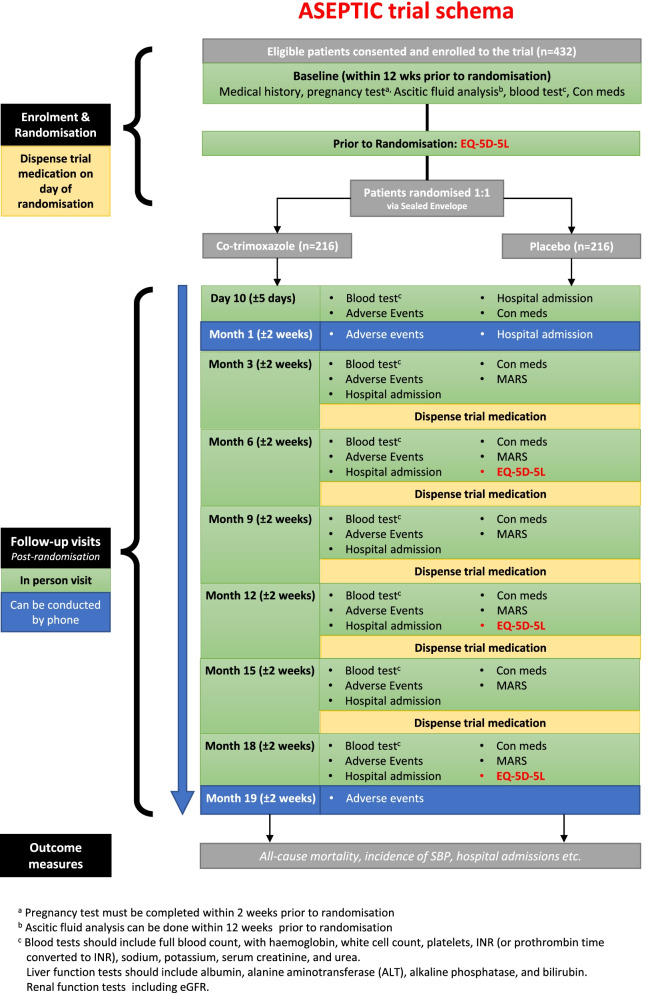
ASEPTIC trial schema

This is a multicentre, randomised, phase 3a, placebo-controlled, double-blind clinical trial. The trial design is displayed in Fig. 1. Patients with cirrhosis and ascites receiving care from at least 30 NHS hospital specialist liver services will be screened using the inclusion and exclusion criteria. We plan to recruit 432 eligible consenting patients that will be allocated in a 1:1 ratio to receive either co-trimoxazole or an identical placebo for 18 months, with a 3-monthly follow-up during this period. Thereafter, post-treatment follow-up will be 6-monthly until the end of the trial, and the maximum possible trial duration for any individual is 48 months.

## Methods: participants, interventions, and outcomes

### Study setting {9}

The trial will take place in the UK at secondary or tertiary care NHS hospitals that frequently manage patients with advanced liver disease. A list of study sites can be found in the Appendix. Patients with cirrhosis and ascites who have been admitted to an inpatient hospital ward or who are attending hospital for an outpatient clinic appointment (including liver transplant waiting list clinics) or abdominal paracentesis on day-case units, will be identified and approached and asked to participate. The nursing and medical staff on the clinical trial team will be appropriately qualified to manage patients with complications of cirrhosis as for routine clinical care.

### Eligibility criteria {10}

The inclusion and exclusion criteria for patient eligibility are listed in Table 1.

**Table 1:** Eligibility criteria for ASEPTIC trial

Inclusion criteria	Exclusion criteria
Patients with cirrhosis of Child–Pugh class B or C and the presence of ascites requiring any diuretic treatment or at least one or more paracentesis within 3 months prior to enrolment	Patients with current or previous SBP (defined as ascitic polymorphonuclear count > 250 cells/mm^3^ with either positive or negative ascitic fluid culture without an evident intra-abdominal surgically treatable source of infection; a white cell count > 500 cell/mm^3^ or positive microbial culture may be considered as evidence of previous SBP if the site PI considers this was in the context of a likely clinical diagnosis of SBP)
At least 18 years of age	Patients receiving palliative care with an expected life expectancy of < 8 weeks
Documented informed consent to participate	Allergy to co-trimoxazole, trimethoprim, or sulphonamides
	Pregnant or lactating mothers
	Patient enrolled in a clinical trial of investigational medicinal products (IMPs) that would impact their participation in the study
	Patients with serum potassium > 5.5 mmol/L related to pre-existing kidney disease which cannot be reduced^a^
	Patients receiving antibiotic prophylaxis (except rifaximin)^a^
	Patients with long-term ascite drains^a^
	Women of child-bearing potential and males with a partner of child-bearing potential without effective contraception for the duration of the trial treatment
	Patients with pathological blood count changes^a^: haemoglobin < 70 g/L, granulocytopaenia defined as absolute neutrophil count < 500 cells/µL, and/or severe thrombocytopaenia with platelets < 30 × 10^9^/L
	Patients with severe renal impairment, with eGFR < 15 mL/min
	Patients with skin conditions: exudative erythema multiforme, Stevens-Johnson syndrome, toxic epidermal necrolysis, and drug eruption with eosinophilia and systemic symptoms
	Patients with congenital conditions: congenital glucose-6-phosphate dehydrogenase deficiency of the erythrocytes and haemoglobin anomalies such as Hb Köln and Hb Zürich
	Patients with acute porphyria
	Any clinical condition which the investigator considers would make the patient unsuitable for the trial

^a^It is common for these investigations to change in patients with cirrhosis, and long-term ascitic drains may be removed. Patients may be rescreened for eligibility if this occurs

Originally, ascitic protein < 2 g/dL was proposed as an inclusion criterion for this trial; however, in the pilot phase, we found a low number of cases of low ascitic protein (38 out of 224 patients), despite only screening patients with refractory ascites and advanced liver disease. Also, there are no large-scale UK data concerning ascitic protein values and the use of ascitic protein in stratifying SBP risk remains contentious [13, 14]. Since it is well recognised that all patients with cirrhotic ascites are at high risk of bacterial infection, it was decided to change the inclusion criteria to persistent ascites without the need for an ascitic protein level. The intention of this amendment was to both increase the number of eligible patients for recruitment and still include patients at the greatest risk of SBP mortality. At the time of this amendment, 15 patients had been recruited. Sub-group analysis will be performed to examine the outcomes in patients with an ascitic protein content ≥ and < 2 g/dL.

The original eligibility requirement regarding the presence of ascites included the condition “despite 3 months of standard treatment”. Feedback from our sites described this time period requisite as a significant limitation to recruitment, and this was formally amended to “patients with cirrhosis of Child–Pugh class B or C and the presence of ascites requiring any diuretic treatment or at least one or more paracentesis within 3 months prior to enrolment”. This enables the recruitment of patients hospitalised with ascites shortly prior to their discharge.

### Who will take informed consent? {26a}

A participant information sheet (PIS) will be given to and discussed with potential patient recruits before consent is sought. Written informed consent will be obtained by appropriately qualified nursing or medical staff who are delegated at each site and have received protocol-specific training.

### Additional consent provisions for collections and use of participant data and biological specimens {26b}

Eligible patients who give their written informed consent will be investigated with baseline blood tests, ascitic fluid sample for white cell count and/or protein level, and pregnancy test if applicable.

### Interventions

#### Explanation for the choice of comparators {6b}

Co-trimoxazole was chosen as the antibiotic for this trial because it has an existing evidence base in SBP prophylaxis and is well tolerated and inexpensive [21–25]. There is a substantial literature on its use to prevent infection in patients living with human immunodeficiency virus (HIV) in resource-poor settings and little evidence of emergent microbial resistance to it [26–28]. Quinolones (e.g. ciprofloxacin or norfloxacin) were also considered as these have been shown to be effective in 2 meta-analyses that included primary prophylaxis trials to reduce the risk of SBP and mortality [4, 5], and they are commonly prescribed for secondary prophylaxis against SBP [15]. However, norfloxacin is not available in the UK, and other systemically absorbed quinolones have been associated with significant rates of antimicrobial resistance and *C. difficile* diarrhoea [29–34]. Rifaximin was not chosen because of its higher cost and widespread use for hepatic encephalopathy in the UK, which would have limited the number of eligible patients and/or risked significantly confounding analyses.

Current EASL guidance (2018) states that norfloxacin prophylaxis should be stopped in patients with long-lasting improvement of their clinical condition and disappearance of ascites but includes no guidance on the duration of therapy in those with persisting ascites [15]. Our trial treatment period of 18 months will test whether there is a beneficial effect on mortality, if there is a waning in efficacy over time, and whether the intervention impacts antimicrobial resistance.

The comparator is placebo, which is an acceptable option as there is no UK consensus regarding the necessity for prescribing primary prophylaxis in SBP and the majority of clinicians who responded to our national survey did not routinely do so.

#### Intervention description {11a}

The investigational medicinal product (IMP) is either one capsule of co-trimoxazole 960 mg once daily or an identical capsule containing placebo once daily. A bottle containing the IMP will be dispensed to patients via the clinical trials pharmacy department at their respective sites to coincide with randomisation and as close to the patient’s 3-monthly follow-up visits as possible. Patients will be instructed to start taking the IMP either as close to the randomisation date as possible (for those recruited in an outpatient setting) or on discharge from the hospital (for those recruited as inpatients).

#### Criteria for discontinuing or modifying allocated interventions {11b}

The IMP may be temporarily stopped if the patient experiences any severe adverse reactions or if, in the opinion of the site principal investigator (PI) or delegate, it is necessary. The patient will be encouraged to remain in the trial and continue to attend follow-up visits. The patient may be re-challenged following stopping of the intervention for at least a week if the PI or delegate considers this safe. The IMP will be adjusted in the following specific scenarios:

- Renal dysfunction: if eGFR falls to < 30 mL/min whilst taking the IMP, dosing will be reduced to one capsule on alternate days and will be increased back to one capsule once daily If eGFR returns to > 30 mL/min. If eGFR reduces to < 15 mL/min whilst taking the IMP, it will be stopped. If the eGFR returns to > 15 mL/min but remains < 30 mL/min, the IMP will be restarted at alternate day dosing but will not be increased to once-daily dosing.
- Hyperkalaemia: if serum potassium rises to ≥ 6 mmol/L, the IMP will be stopped and re-introduced when the site PI considers it safe. If hyperkalaemia ≥ 6 mmol/L occurs twice or more whilst taking the IMP, it will be reduced to alternate day dosing on re-introduction for the remainder of the study.
- Thrombocytopaenia: if the platelet count falls to < 50 × 10^9^/L with active bleeding, to < 30 × 10^9^/L without active bleeding, or in a manner that concerns the PI or clinical team regarding increased risk of bleeding, the IMP will be stopped until counts are ≥ 30 × 10^9^/L, any bleeding has resolved, and it is deemed safe to do so.
- Non-SBP-related hospital admission: the IMP should be stopped upon admission to the hospital and restarted when safe to do so, on or soon after discharge.
- Recovery from ascites: if ascites resolves for > 6 months without diuretic medications, patients will stop taking the IMP as their health status has improved.

The IMP will be stopped in the following scenarios:

- Stevens-Johnson syndrome.
- Hospital admission with SBP. As per routine clinical practice, patients will be commenced on long-term antibiotics as secondary prophylaxis following treatment of SBP.
- Any significant medical reason deemed by the local PI.
- Any suspected unexpected serious adverse reaction (SUSAR) event.

Every effort will be made to ensure patients withdrawn from the trial treatment continue to be followed up as per the protocol until the trial end.

#### Strategies to improve adherence to interventions {11c}

Patients will be educated about the possible risks of non-adherence. Treatment adherence will be assessed at regular intervals using the Medication Adherence Report Scale (MARS) questionnaire [35].

#### Relevant concomitant care permitted or prohibited during the trial {11d}

Concomitant care with long-term antibiotic prophylaxis is not permitted, except for rifaximin prescribed for hepatic encephalopathy. Patients will be stratified according to their use of rifaximin at randomisation and whether they are presently on the waiting list for a liver transplant. As hyperkalaemia is a known potential side effect of co-trimoxazole, caution will be taken in patients taking other medications that can cause hyperkalaemia: ACE inhibitors, angiotensin receptor blockers, and potassium-sparing diuretics. Patients at increased risk of renal dysfunction and/or hyperkalaemia, namely those with existing kidney dysfunction and those taking spironolactone or amiloride, are not eligible if the PI judges this risk cannot be managed with the adjustment of diuretics.

#### Provisions for post-trial care {30}

Other medical care during and post-trial will be standard medical care.

### Outcomes {12}

The primary outcome is overall survival during follow-up.

The secondary outcomes are as follows:

1. Time to the first incidence of SBP
2. Hospital admission rates at 18 months
3. Incidence of *C. difficile*-associated diarrhoea at 18 months
4. Incidence of infections other than SBP with hospital admission at 18 months
5. Incidence of other cirrhosis-related events (e.g. variceal haemorrhage) at 18 months
6. Incidence of renal dysfunction with creatinine > 133 µmol/L (1.5 mg/dL) at any point during hospital admission
7. Incidence of anti-microbial resistance at 18 months
8. Incidence of liver transplantation at 18 months
9. We will explore the potential impact of alcohol cessation and recidivism rates in those with alcohol as a cause of cirrhosis and any interaction with treatment
10. Progression of liver disease assessed by an increase in Model for End-Stage Liver Disease (MELD) score between baseline and end of trial follow-up
11. Safety and treatment-related serious adverse events
12. Treatment adherence (assessed by MARS questionnaire)
13. Incidence of resolution of ascites with diuretic treatment not required for 6 months at 18 months
14. Incidence of transjugular intrahepatic portosystemic shunt (TIPS) insertion at 18 months
15. Health-related quality of life assessed using the EQ-5D-5L Questionnaire
16. Health and social care resource use assessed using Hospital Episode Statistics (HES) database
17. Mean incremental cost per quality-adjusted life year (QALY) gained

#### Participant timeline {13}

The assessments and interventions required at screening, randomisation, and follow-up visits are outlined in Table 2. Eligible patients will be randomised using the secure randomisation website, Sealed Envelope® (www.sealedenvelope.com), which generates anonymised kit codes for trial staff to identify the correct trial medication for the patient, whilst maintaining blinding. Patients should start taking the trial medication as close as possible to the randomisation date (for those enrolled as outpatients) or as soon as they are well enough after discharge from the hospital (for those enrolled as inpatients). Follow-up visits by contact at the outpatient appointment will be on day 10, then at outpatient appointments or by telephone at month 1 and 3-monthly thereafter for 18 months. At month 19, there will be an end-of-study safety telephone call as this marks the end of the treatment period for the trial and denotes the minimum follow-up period. Patients that finish the treatment period prior to the end of the trial will be followed up 6-monthly until the end of the trial.

**Table 2:** Trial schedule of interventions and assessments

Visit number	Visit 1		Visit 2	Visit 3	Visit 4	Visit 5	Visit 6	Visit 7	Visit 8	Visit 9	Safety call	Long term follow-up
	(< 12 weeks from randomisation)	Month 0	Day 10	Month 1	Month 3	Month 6	Month 9	Month 12	Month 15	Month 18	Month 19	Every 6 months from month 24 to the end of the trial
	Screening	Randomisation	Follow-up									
Eligibility screening	X											
Informed consent	X											
Medical history	X											
Pregnancy test (*if applicable*)	X^a^											
Ascitic fluid analysis	X^b^											X
Blood tests, liver and renal function tests^c^	X		X^e^		X	X	X	X	X	X		X
Randomisation		X										
Adverse events review			X	X	X	X	X	X	X	X	X	X
Hospital admission review			X	X	X	X	X	X	X	X		X
Concomitant medication review	X		X		X	X	X	X	X	X		X
MARS Questionnaire					X	X	X	X	X	X		
EQ-5D-5L Questionnaire		X				X		X		X		
Dispense trial medication^d^		X			X	X	X	X	X			

^a^Pregnancy test must be completed within 2 weeks prior to randomisation

^b^Ascitic fluid analysis can be done within 12 weeks prior to randomisation

^c^Blood tests should include full blood count, with haemoglobin, white cell count, platelets, INR (or prothrombin time converted to INR), sodium, potassium, serum creatinine, and urea. Liver function tests should include albumin, alanine aminotransferase (ALT), alkaline phosphatase, and bilirubin. Renal function tests including eGFR

^d^At each indicated visit, one bottle of 100 capsules of trial medication is dispensed to the patient (either co-trimoxazole or placebo capsules)

^e^Visit 2 is to monitor the safety of the patient and to make any dose modifications to concomitant medications if needed

#### Sample size {14}

For overall survival as the primary outcome, we anticipate a hazard ratio (HR) of 0.62 and calculated that 432 patients will be required to generate 187 events, incorporating a 10% cumulative probability of loss-to-follow-up by the end of the study. Guided by the Kaplan–Meier 18-month survival estimate amongst patients with cirrhosis and ascites in the control arm of the ANSWER trial [30], and assuming an exponential survival distribution, we anticipate 66% survival in the control arm at 18 months. In the experimental arm, we expect 77% of patients surviving at 18 months. The recruitment will be uniform over a period of 2.5 years, and the follow-up time will be 18 months minimum with a maximum potential follow-up of 48 months. This calculation was based on a two-sided 5% type 1 error rate and 90% power. The primary event rate overall (blinded to allocation) will be monitored by the trial steering committee, and follow-up may be extended beyond the planned period to achieve the required number of events, with any extension agreed with the funder. Disruption to the trial schedule owing to the COVID-19 pandemic may necessitate an extension of the recruitment period.

#### Recruitment {15}

Both outpatients and inpatients will be recruited from at least 30 secondary or tertiary NHS hospitals that frequently manage patients with advanced liver disease. Outpatients will be screened when attending hepatology or liver transplant waiting list clinic appointments, or day case units for elective ascitic paracentesis. The recruitment period will be 30 months.

### Assignment of interventions: allocation

#### Sequence generation {16a}

The independent, online, computer-generated randomisation service, Sealed Envelope® (www.sealedenvelope.com), will be used to minimise allocation bias. Randomisation will use a minimisation algorithm incorporating a random element, stratifying by active participation on the liver transplant waiting list, rifaximin prescription at enrolment, and centre. To ensure a maximum balance is achieved across the stratification factors, minimisation will be carried out on these factors separately. In mid-2022, we submitted an amendment to include alcohol as a significant cause of cirrhosis and active alcohol consumption at randomisation as stratification variables following review by our oversight committees.

#### Concealment mechanism {16b}

The Sealed Envelope® software allows concealed allocation. A single labelled bottle with a unique kit code of the IMP will be dispensed following randomisation and at each subsequent 3-monthly follow-up visit.

#### Implementation {16c}

The responsibility for confirming patient eligibility and prescribing the IMP lies with the site PI or delegated clinicians. Eligibility decisions will be made in line with the approved protocol. Other clinicians employed at the same clinical site (including trial nurses who are registered independent prescribers) may enrol and prescribe trial treatments to patients only if they have received appropriate training on the trial and appear on the trial delegation log, approved by the PI.

### Assignment of interventions: blinding

#### Who will be blinded {17a}

Trial patients and all trial staff including clinicians and dispensing pharmacists will be blinded. The co-trimoxazole and placebo capsules will appear identical.

#### Procedure for unblinding {17b}

There will be no unblinding unless considered important for the patient’s care as assessed by the attending clinicians. If emergency unblinding is deemed necessary, this can occur at any time through, Sealed Envelope®.

### Data collection and management

#### Plans for assessment and collection of outcomes {18a}

Each patient will be given a unique trial patient identification number (PIN). Data will be collected using paper case report forms (CRFs) and will also be entered directly into an electronic data capture database (InferMed MACRO). Clinical outcomes will be logged by the trial staff. Validated questionnaires (e.g. MARS and EQ-5D-5L) will be used to collect data on certain outcomes.

#### Plans to promote participant retention and complete follow-up {18b}

The trial follow-up is designed to align with regular clinical follow-up appointments to minimise the additional burden for participants thus promoting retention. Patients that finish the treatment period prior to the end of the trial will be followed up 6-monthly until the end of the trial.

#### Data management {19}

Identification, screening, and enrolment logs will be kept at each trial site in a locked cabinet in a secured room and/or electronically. Electronic data will be stored on secure servers based at the lead organisation. The database will be password-protected and only accessible to specified trial members. After completion of the trial, these logs will be archived and stored securely by the sites for a minimum of 5 years. All data will be handled in accordance with the Data Protection Act 2018.

#### Confidentiality {27}

All data will be handled in accordance with the Data Protection Act 2018.

#### Plans for collection, laboratory evaluation, and storage of biological specimens for genetic or molecular analysis in this trial/future use {33}

Not applicable as no biological specimens for genetic or molecular analysis will be collected as part of this trial.

## Statistical methods

### Statistical methods for primary and secondary outcomes {20a}

A detailed statistical analysis plan will be written prior to the first unblinded analysis and approved in advance by the trial steering committee. The main analyses will be conducted following the intention-to-treat principle. For the primary outcome, Kaplan–Meier survival curves will be compared using the log-rank test, according to the allocated treatment in an intention-to-treat manner. An unadjusted Cox proportional hazards model will be fitted, and the unadjusted hazard ratio and 95% confidence interval will be presented. An adjusted Cox model will then be fitted, adjusting for the stratification factors (active participation on the liver transplant waiting list, rifaximin prescription at enrolment, alcohol as a cause of cirrhosis, and active alcohol consumption at randomisation if the amendment submitted in June 2022 is successful) by including them as covariates in the model. All statistical tests will use a two-sided *p*-value of 0.05.

If there is evidence of non-proportional hazards, the life expectancy difference and life expectancy ratio between the two arms will be presented [36]. The stratified log-rank test will be reported in this instance as well, and the odds ratio for death at 18 months will be calculated using logistic regression.

Binary secondary outcomes will be analysed using logistic regression, and continuous outcomes using linear regression adjusting for baseline values (i.e. ANCOVA). Time to the first incidence of SBP will be presented with Kaplan–Meier curves and compared between the arms using the log-rank test. Serious adverse event rates will be presented by treatment arm and grade. Quality of life scores will be compared using a hierarchical linear regression model.

### Interim analyses {21b}

There are no planned interim analyses. Regular reports regarding patient safety, death, and SBP events will be prepared for IDMC, who will have untrammelled access to study data in order to ensure participants are not placed at avoidable risk. The IDMC processes are described in a separate charter.

### Methods for additional analyses (e.g. subgroup analyses) {20b}

The results on the primary efficacy outcome will be presented according to the levels of the stratifying variables used in the randomisation process. Interaction terms between each of the variables below and treatment will be added in turn to the primary analysis model to investigate whether the treatment effect differs according to the levels of these factors. We will report the treatment effect estimates in the subgroups and the *p*-value of the interaction tests.

The factors for subgroup analysis are as follows:

- Rifaximin prescription at randomisation
- Active participation on liver transplant list at randomisation
- Gender
- Ascitic fluid protein count (above or below the cut-off of 2 g/dL)
- Long-term ascites (ascites persistent for more than 3 months at enrolment)

#### Health economic analysis

We aim to calculate the mean incremental cost per QALY gained from using co-trimoxazole to prevent SBP and improve overall survival in cirrhosis patients. The primary analysis will be a within-trial intention-to-treat analysis. Because an 18-month follow-up is considered enough time to capture all important drivers of the cost-effectiveness of the intervention, no economic modelling will be required. Health-related quality of life will be measured using the EQ-5D-5L, which will be collected at baseline and every 6 months during the 18-month follow-up for each patient. Utility scores will be calculated using UK-specific tariffs [37]. QALYs will be calculated as the area under the curve adjusting for baseline differences and minimisation factors as will be specified in the health economic analysis plan. Cost savings are anticipated to result from the prevention of hospital re-admissions and a reduction in other healthcare resource use. Resource use will be valued from the health and social care services perspective. The data will include hospital admissions (including length of stay and type of ward), cost of co-trimoxazole, day-case visits, any tests undertaken, outpatient attendances, primary care contacts, A&E visits, and concomitant medications. Unit costs will be obtained from publicly available sources such as the British National Formulary [38] and Unit Costs of Health and Social Care [39]. We will report descriptive statistics for all health and social care resource use at 18 months for both arms. 95% confidence intervals for the difference in costs between the two arms will be based on the bootstrapped results adjusting for variables specified in the analysis plan. Bootstrapped results will be also used to report the incremental cost per QALY gained. Cost-effectiveness acceptability curves will be constructed to report the probability that co-trimoxazole is cost-effective compared to placebo for a range of values of the cost-effectiveness threshold recommended by NICE [40].

### Methods in analysis to handle protocol non-adherence and any statistical methods to handle missing data {20c}

The primary analysis will be conducted with the intention to treat principle, without imputation. We will undertake supportive analyses to consider the potential effects of missing data on the primary objective, using threshold analysis (where intervention group subjects who are missing are considered to have died the day after their last contact, and similarly control group subjects will be censored on the day after their last contact).

### Plans to give access to the full protocol, participant-level data, and statistical code {31c}

Enquiries may be addressed to ctu.aseptic@ucl.ac.uk.

### Oversight and monitoring

#### Composition of the coordinating centre and trial steering committee {5d}

Trial oversight is delegated by the trial sponsor, UCL, to the UCL Comprehensive Clinical Trials Unit (CCTU). The trial team assists with the trial design, coordination and day-to-day operational issues in the management of the trial. The Trial Management Group (TMG) assists with the design, coordination, and strategic management of the trial. The TMG is composed of the CI, expert clinicians (hepatologists, microbiologists), PIs, clinical project managers, trial managers, statisticians, data managers, and health economists.

The Independent Trial Steering Committee (TSC) is responsible for the oversight of the trial to safeguard the interests of trial patients. The TSC is composed of independent expert clinicians, independent members and patient representatives, non-independent member clinicians, and observers.

#### Composition of the data monitoring committee, its role, and reporting structure {21a}

The Independent Data Monitoring Committee (IDMC), comprised of a clinician with expertise in liver disease, a clinical trialist, and a statistician, is responsible for safeguarding the interests of trial participants. They are the only oversight body that can access unblinded accumulating comparative data.

#### Adverse event reporting and harms {22}

Definitions of harm of the EU Directive 2001/20/EC Article 2, based on the principles of ICH Good Clinical Practice guidelines, apply to this trial. All adverse events (AEs) and serious adverse events (SAEs) occurring during the trial observed by the investigator or reported by the patient will be recorded in the patient’s medical records as per standard practice and, if applicable, on the appropriate case report form. All related SAEs should be notified to the CCTU immediately and within 24 h of the investigator becoming aware of the event. All unrelated SAEs will still be collected but do not require expedited reporting.

#### Frequency and plans for auditing trial conduct {23}

During the course of the trial, selected sites will be monitored (on-site or remote) to ensure quality assurance in that the site is adhering to the trial protocol, NIHR Good Clinical Practice (GCP) guidelines and regulations, ensure patient safety and that the data collected is accurate. In addition, the sites will be centrally monitored where study data will be regularly checked for any anomalies.

#### Plans for communicating important protocol amendments to relevant parties (e.g. trial participants, ethical committees) {25}

We have two liver patient group representatives on our TSC and have the support of the British Liver Trust. In addition, the TSC includes members who have clinical expertise and any proposed changes made to the protocol will be raised through meetings or via email discussions prior to submitting for REC/HRA and MHRA approvals.

#### Dissemination plans {31a}

The results will be promoted via peer-reviewed publication to maximise the chances of adoption into clinical practice, regardless of the direction of effect on outcomes. Research findings will also be presented at conferences and seminars. Plain language summaries of the research findings will be written and disseminated to trial participants and the wider public. Publicity and engagement with the public and healthcare users will be supported by the British Liver Trust, the Primary Sclerosing Cholangitis Trust, the Liver Patients’ Transplant Consortium, and the UCL Public Engagement Unit.

## Discussion

ASEPTIC is a multicentre RCT that aims to evaluate the efficacy, safety, and cost-effectiveness of co-trimoxazole for primary prophylaxis of SBP in patients with cirrhosis and ascites. The current clinical guidelines in this area are inconsistent and those that recommend primary prophylaxis only do so in the context of low ascitic protein levels, despite the evidence underlying this being contested [6, 15, 18, 41]. Our survey of clinicians confirmed the lack of consensus in clinical practice and further highlighted the need for a robust RCT to inform practice (unpublished data). Co-trimoxazole was chosen as it is a widely prescribed and relatively inexpensive antibiotic with a favourable safety profile.

The strengths of the trial design include robust mechanisms of randomisation, blinding, and the use of placebo. The inclusion criteria are deliberately broad to accurately represent the population of patients with advanced liver disease who use secondary and tertiary hepatology services in the UK. This should ensure trial outcomes are as relevant and applicable to this patient group as possible in clinical practice, and thus our primary analysis will present a treatment policy estimand. Pragmatic amendments have also been made to maximise recruitment, such as allowing recruitment of both inpatients and outpatients, and removing the cut-off of ascitic protein level < 2 g/dL as an inclusion criterion. Furthermore, mitigations were added in response to the challenges of the COVID-19 pandemic, for example, the option of delivering the IMP to patients’ homes by courier if required.

One major challenge of running long-term interventional trials in patients with advanced liver disease is the high rate of complications, hospital admissions, and mortality in this population. Recent large-scale clinical trial data from the ANSWER study (*n* > 400) demonstrated an 18-month mortality rate of > 20% for patients with cirrhosis and persistent ascites [30]. Our trial is recruiting a very similar cohort to ANSWER; therefore, it has been possible to use its results as a guide to power our study using overall survival as a primary outcome. Furthermore, mortality is easier to record accurately compared with infection, and ultimately the clinical trajectory that antibiotic prophylaxis aims to prevent is infection causing organ failure leading to death. If proven effective, primary antibiotic prophylaxis could also provide a bridge to liver transplantation for some patients.

Large-scale clinical trials are rarely performed in patients with cirrhosis and ascites, and there are no treatments that extend life in these patients outside of liver transplant. Therefore, the results of this large, multicentre RCT are anticipated to have a major impact on informing evidence-based practice for the benefit of patients with advanced liver disease.

## Trial status

The ASEPTIC trial began recruitment in September 2019 and recruitment is ongoing. Due to the COVID-19 pandemic, recruitment was paused, which affected trial timelines. The new anticipated recruitment end date will be mid-2023.

## Data Availability

The main outcomes will be published in a peer-reviewed journal. Unidentifiable participant data will be presented in the trial results. The full study protocol and statistical analysis plans can be requested by email to ctu.aseptic@ucl.ac.uk.

## Appendix

List of sites recruiting patients at the time of writing (20 June 2022), in alphabetical order.

1. Aintree University Hospital
2. Basildon University Hospital
3. Bedford Hospital
4. Bristol Royal Infirmary
5. Glasgow Royal Infirmary
6. Gloucester Royal Hospital
7. Hull Royal Infirmary
8. James Paget University Hospital (Great Yarmouth)
9. John Radcliffe Hospital (Oxford)
10. Kettering General Hospital
11. King’s College Hospital (London)
12. Luton & Dunstable Hospital
13. Newcastle Freeman Hospital
14. Ninewells Hospital and Medical School (Dundee)
15. Nottingham University Hospital
16. Queen Alexandra Hospital, Portsmouth
17. Queen Elizabeth Hospital, Gateshead
18. Queen Elizabeth University Hospital, Glasgow
19. Royal Berkshire Hospital (Reading)
20. Royal Derby Hospital
21. Royal Free London
22. Royal Infirmary of Edinburgh
23. Royal Liverpool University Hospital
24. Royal London Hospital (Barts Health)
25. Royal Sussex County Hospital
26. St George’s Hospital (London)
27. St James University Hospital, Leeds
28. University College London Hospital
29. University Hospital North Durham
30. University Hospital of North Tees
31. University Hospital of Wales (Cardiff)
32. University Hospital of Coventry and Warwickshire
33. Whittington Hospital

## Abbreviations

AASLD: American Association for the Study of Liver Disease
AE: Adverse event
ALT: Alanine aminotransferase
ANSWER: Human Albumin for the treatmeNt of aScites in patients with hEpatic ciRrhosis
ASEPTIC: Primary Antibiotic prophylaxis using co-trimoxazole to prevent SpontanEous bacterial PeritoniTIs in Cirrhosis
BSG: British Society of Gastroenterology
*C. difficile*: *Clostridium difficile*
CCTU: Comprehensive Clinical Trials Unit
COVID-19: Coronavirus disease 19
CRF: Case report form
EASL: European Society for the Study of the Liver
GCP: Good Clinical Practice
HES: Hospital Episode Statistics
HIV: Human immunodeficiency virus
IDMC: Independent Data Monitoring Committee
IMP: Investigational medicinal product
MARS: Medication Adherence Report Scale
MELD: Model for End-Stage Liver Disease
MHRA: Medicines and Healthcare products Regulatory Agency
NHS: National Health Service
NICE: National Institute of Health and Care Excellence
NIHR: National Institute for Health and Care Research
PI: Principal investigator
PIN: Patient identification number
PIS: Patient information sheet
QALY: Quality of life year
RCT: Randomised controlled trial
SAE: Serious adverse event
SBP: Spontaneous bacterial peritonitis
SUSAR: Suspected unexpected serious adverse reaction
TIPS: Transjugular intrahepatic portosystemic shunt
TMG: Trial Management Group
TSC: Trail Steering Committee
UCL: University College London
